# Characterisation of antimicrobial usage in Danish pigs in 2020

**DOI:** 10.3389/fvets.2023.1155811

**Published:** 2023-04-25

**Authors:** Pedro Moura, Marianne Sandberg, Birgitte Borck Høg, João Niza-Ribeiro, Elisabeth Okholm Nielsen, Lis Alban

**Affiliations:** ^1^National Food Institute, Technical University of Denmark, Lyngby, Denmark; ^2^Departamento de Estudo de Populações, ICBAS, Instituto de Ciências Biomédicas Abel Salazar, Universidade do Porto, Porto, Portugal; ^3^Epidemiology Research Unit (EPIUnit), Instituto de Saúde Pública da Universidade do Porto, Porto, Portugal; ^4^SEGES Innovation, Copenhagen, Denmark; ^5^Department for Food Safety, Veterinary Issues and Risk Analysis, Danish Agriculture and Food Council, Copenhagen, Denmark; ^6^Department of Veterinary and Animal Sciences, University of Copenhagen, Frederiksberg, Denmark

**Keywords:** antimicrobials, surveillance, consumption, usage, stewardship, Denmark, pigs, one-health

## Abstract

**Introduction:**

Denmark is one of the world’s largest exporters of pigs and pig meat, so the sector plays an important role in the national antimicrobial use (AMU). The Danish government has run antimicrobial stewardship programs in collaboration with the pig industry for more than 25 years. These have resulted in substantial overall reductions in total AMU and limiting the use of fluoroquinolones, the 3rd and 4th generation cephalosporines and the polymyxin colistin. To understand where further reductions in AMU could take place, it is necessary to investigate which antimicrobials are being used, how, and for which reasons.

**Materials and methods:**

We characterized the AMU in the Danish pig sector in 2020, providing new analytical insights based on data retrieved from the VetStat database. The AMU data were segmented into classes, routes of administration, treatment indications and age groups, and interpreted as an outcome of the interventions taken. We evaluated the current AMU regarding choice of antimicrobial class. Moreover, we discussed how to further improve the antimicrobial stewardship in Danish pig production to achieve additional reductions without jeopardizing animal welfare. Where relevant, two pig veterinary specialists were consulted.

**Results:**

In 2020, 43.3 mg antimicrobials per population correction unit (PCU) were ascribed to the Danish pig sector. There was practically no use of fluoroquinolones, 3^rd^ and 4^th^ generation cephalosporins and polymyxins. Weaners related to 45% of the total AMU in pigs when measured in tonnes and 81% when measured in defined animal daily doses, of these 76% were ascribed to gastrointestinal indications and overall, 83% were administered perorally.

**Conclusion:**

To enable further reductions in AMU, it should be investigated how and when to replace group treatments (e.g., all animals in section or a pen) with individual treatments. Moreover, prevention of disease and promotion of animal health should be prioritized, e.g., through focus on feed, vaccination, biosecurity, and disease eradication.

## 1. Introduction

In modern medicine, antimicrobials (AM) constitute fundamental instruments for the control of bacterial infectious diseases. However, when AM are used, a natural evolutionary selection is triggered, selecting the most well-adapted bacteria that can obtain, express, and propagate genes more fitting to survival than other bacteria (1). Therefore, the use of AM should be prudent, especially of those that have been defined as critically important by the European Medicines Agency (EMA) (2), so the last line of defence against infections is maintained.

The World Health Organization (WHO) has recognized the fight against AM resistance (AMR) as one of the most important challenges that humanity will have to face in the present decade (3). Given that AMR genes can circulate in any direction within and between a global and complex system composed of the environment, humans and animals, a coordinated One Health (OH) approach is crucial to comprehend the problem (4). Prudent use of AM will ensure that humans and animals in need of AM treatment can be treated not just now but also in the future (5). However, animal welfare may be challenged, if animals with severe infections are not treated with AM. To take both these concerns into account, the Danish pig industry has developed the approach called “As little as possible, but as much as necessary” (6), where improving animal health and, consequently, reducing the need for AMU are central to antimicrobial stewardship.

Antimicrobial stewardship can be defined as “A coherent set of actions which promotes using antimicrobials responsibly” (7), with the primary goal being “to optimize clinical outcomes while minimizing unintended consequences of antimicrobial use” (8). Monitoring both AMU and the development of AMR allows the interpretation of patterns and trends of AMU, which can be related to the emergence of AMR, enabling risk evaluation and management, and therefore constituting the basis of AM stewardship programs (9). AMU data can, within a given time frame, also be used to evaluate the efficacy of control measures implemented, and when the same indicators are applied establish international comparisons (10).

AM stewardship programs across Europe have received international recognition. Examples of good practices can be made out of the Danish pig sector (11), along with the Dutch model where a combination of mandatory and voluntary actions (12) have resulted in a shift from 3rd and 2nd to 1^st^ choice AM compounds in the dairy sector (13). Likewise, the multisectoral voluntary approach implemented in the United Kingdom has resulted in a 50% reduction in overall AMU in the livestock industry from 2014 and 2021, including a 79% reduction in the use of highest-priority critically important antibiotics during the same period (14).

To elucidate whether the AMU and AMR monitoring systems in place and actions taken to combat AMR are effective, it is necessary to evaluate them at regular intervals. Several tools have been developed to help in this (15). In an international network called Convergence in evaluation frameworks for integrated surveillance of AMU and AMR (CoEvalAMR), guidelines have been developed for evaluation along with assessment of different evaluation tools. One of these tools is the Integrated Surveillance System Evaluation (ISSE), which is a conceptual framework for evaluation of the performance and the value of OH integration in surveillance systems for AMU and AMR. According to ISSE, evaluations can be done at different levels such as production of information and expertise, generation of actionable knowledge, influence on decision-making and contributions to desirable outcomes. All this will enable an evaluation of the impact of the decisions made (16).

**Figure 1:**
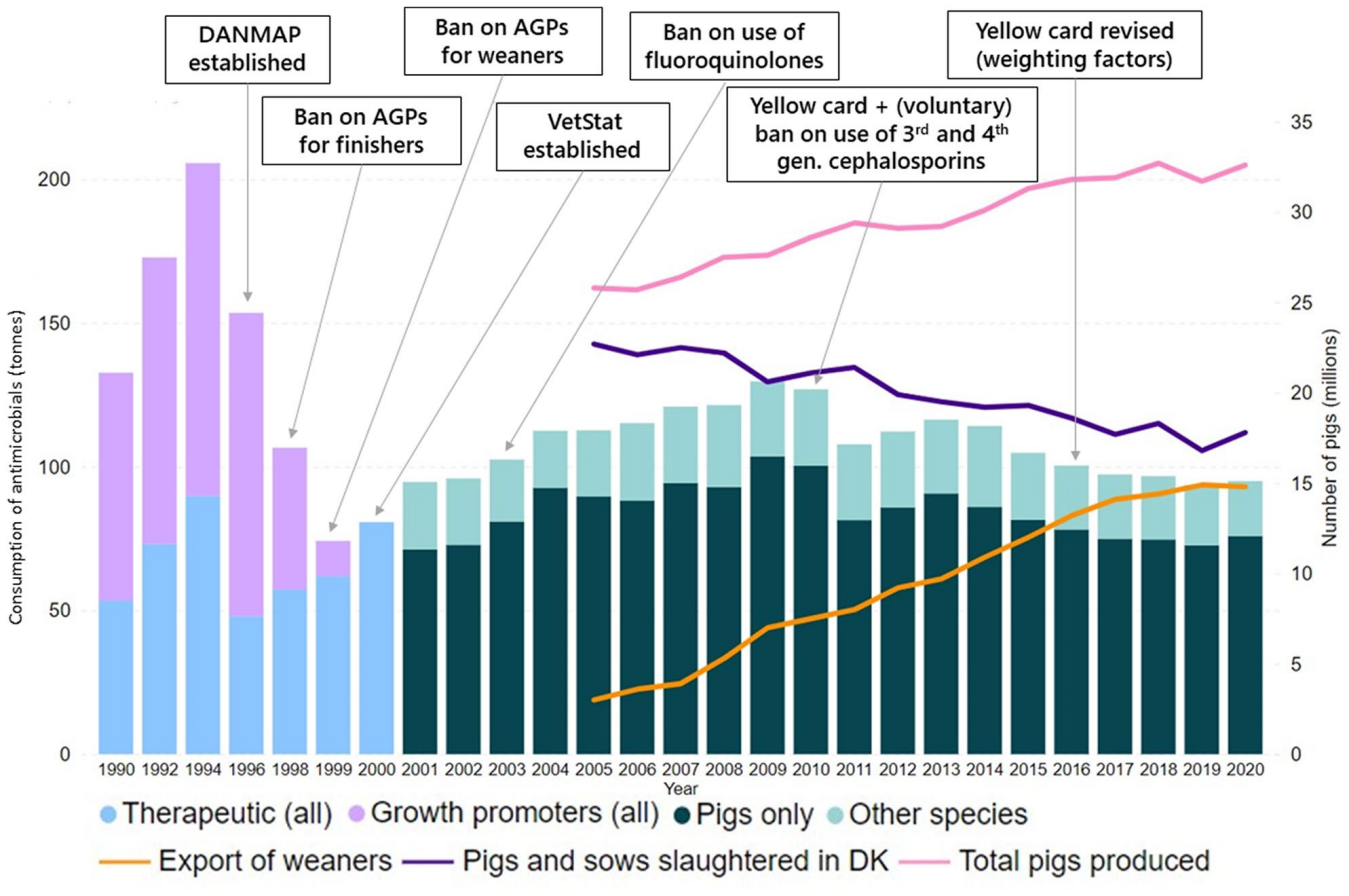
Total amount of AMU in Danish livestock from 1990 to 2000 and in the pig sector from 2001, total number of pigs produced in Denmark since 2005, divided into weaners exported and pigs and sows slaughtered domestically, and risk mitigating initiatives. AGP, Antimicrobial growth promoters; DK, Denmark; DANMAP, Danish Integrated Antimicrobial Resistance Monitoring and Research Program.

Denmark is a “pig country.” In 2020, there were 2,921 active and professional farms with pigs registered; the sector produced 32.6 million pigs, with 17.5 million of these being slaughtered in the country, and 14.8 million were exported as weaners at 30kg of weight (17) as seen in Figure 1, in addition 0,3 million finishers and sows were exported for slaughter. Monitoring of AMU is at the age groups (1) sows and piglets and (2) weaners, and (3) finishers (18). In the Danish pig sector, AMR trends are monitored by indicator *Escherichia coli* isolates obtained from arbitrarily selected caecal samples collected at slaughter, and from fresh, chilled meat collected at retail points, tested in accordance with EU requirements (19). Denmark has been implementing AM stewardship measures for over 25 years as shown in the following:

A national ban on the use of AM as growth promoters came into force in finishers in 1998 and in weaners in 1999, whereas this came into force in the European Union (EU) in 2006 (20), Figure 1. Veterinary advisory service contracts are required for the large pig herds, i.e., with more than 300 sows (21), and there is a limitation of veterinarians’ profits from AM sales (11). Moreover, direct marketing of prescription-only drugs and vaccines to layman is prohibited (22). Since 2001, AMU is reported into the VetStat database, which has been the basis for implementing sector interventions and measuring their impact. Pharmacies are obliged to report the amount of AM that the veterinarians prescribe and specify the target age group and treatment indication. Feed mills similarly report AM-medicated feed sales at farm level, while veterinarians directly report the amounts of AM they prescribe and use in clinical practice (23).

The Danish Integrated Antimicrobial Resistance Monitoring and Research Program (DANMAP) provides a complete and transparent picture of AMU and the occurrence of AMR in bacteria from food animals, food and humans in Denmark. For that reason, DANMAP serves as the basis for implementing evidence-based policies (24), e.g., since 2002, the prescription of fluoroquinolones is antibiogram-dependent (11), and in 2010 a voluntary industry ban on the use of 3rd and 4th generation cephalosporins was introduced in Danish pig production (11).

The Yellow Card initiative, which was established in 2010, sets limits to the acceptable AMU at the individual herd level. Benchmarking figures are defined for the different animal age group, based on the average AMU over the last 9 months, calculated as defined animal daily dosages (DADD) per 100 animals per day. These figures for the individual herd are then compared with national permit limits (20). Originally, these permit limits were defined as twice the national average within the age group. These have been lowered over time. The current thresholds in DADDs are 3.2 for sows and piglets, 4.4 for finishers and 17.2 for weaners (25). Some critically important AMs are weighted by a factor above 1 to reflect the AM’s perceived negative impact on AMR development, and this increases the registered DADD value: i.e. 3^rd^ and 4^th^ generation cephalosporins, colistin and fluoroquinolones have a weighting factor of 10, whereas tetracyclines have a weighting factor of 1.5, while unrestricted AMs, such as penicillins have a weight factor of 1 (20).

To understand the current AMU in the Danish pig sector, it is important to provide historical context to the figures regarding when the different risk mitigating measures were implemented and how the sector developed. As can be seen in Figure 1, the overall AMU in tonnes was reduced by 52% from 1994 when consumption reached its maximum to 2020, despite concurrent growth of the pig sector. AMU per species only became available after the establishment of VetStat.

To combine an optimal clinical effect with the lowest possible adverse impact from the development of AMR, it is crucial to select the appropriate AM. The European Medicines Agency’s EMA’s Antimicrobial Advice *Ad Hoc* Expert Group (AMEG) classification is based on considering the probability and consequences associated with the use of a specific AM regarding the development of AMR, as well as its importance in human medicine, while considering the existence of alternative substances. In this classification: Category A “Avoid” consists of AM that are not licensed for use in animals; Category B substances include 3rd and 4th generation cephalosporins, quinolones and polymyxins, which should be of “Restricted” use in veterinary medicine, as these are critically important substances in human medicine; Category C “Caution” includes AMs for which there are reliable alternatives in human medicine, but few veterinary alternatives; Category D “Prudence” covers AM for use as a first line of treatment, in a prudent way whenever possible (Supplementary Table S1) (2).

In Denmark, prescription guidelines have been released by the Danish Veterinary and Food Administration (DVFA), classifying certain AM substances as first choice (Group 1), as alternatives (Group 2) or of restricted use (Group 3) (26), Supplementary Table S1. As part of that work, risk assessments have been undertaken for selected AMs in pigs, i.e., macrolides (27) and pleuromutilin (28). These guidelines are progressively updated according to new knowledge about AMR development. Preventing the occurrence of disease by investing in use of vaccination is also an important health promoting initiative, which ultimately comes down to each farmer’s decision (29). As can be seen, in Supplementary Figure S1, vaccination sales for some of the most common pathogens have gone up in recent years.

The overarching question is: What is the current state in Denmark with respect to AMU after more than 25 years of interventions to combat AMR development?

The detailed objectives of this paper are to:

- Characterize Danish AMU in the three age groups: weaners; finishers; sows and piglets in 2020.
- Based upon the figures produced, discuss the current AMU in Danish pig production with respect to risk of development of AMR, where AMU is seen as a driver for AMR. Discuss whether it would be feasible to further improve the antimicrobial stewardship through reduction of AMU by moving usage from oral to parenteral treatment or alter use patterns, without jeopardizing animal welfare.

## 2. Materials and methods

### 2.1. Data

The data regarding AMU in the Danish pig sector in 2020 originated from Vetstat and consisted of the data used in 2020 DANMAP report. The same data, which also encompass the national antimicrobial sales figures, are reported annually to the European Surveillance of Veterinary Antimicrobial Consumption (ESVAC) report. This report contains the total sales of antimicrobial agents for veterinary use in livestock production from 31 European countries (30). ESVAC figures are published using a statistic called mg per population correction unit (PCU), hence the use in milligrams (mg) of active substance is normalized by a standardized estimate of the national animal biomass (30).

To calculate this statistic specifically for the Danish pig sector, the figure describing national AMU sales was taken from the 2020 DANMAP Report: 7.59 × 10^10^ mg of AM active substance sold (20). For PCU, a figure of 1.754 × 10^9^ kg, calculated according to EMA’s directives and originating from the European database of sales of veterinary antimicrobial agents was used (31).

AMU measured in defined animal daily dosages (DADD) per licensed medicinal product had previously been calculated by the DANMAP team and these figures were made available for this analysis. DADD is the average maintenance dose per day for the main indication of a drug in the appropriate animal species. It is calculated using the following formula:


(Formula 1) $$DADD=\frac{mg\;ofactivesubstance}{\begin{array}{l} \;DANMAPdosage\;per\;kg\;ofbody\\ weight\times standardweightofanimal\;\\ \;\;\;\;\;\;\;\;\;\;\;\;\;\;\;\;\;\;\;\;\;\;\;\;\;\;\;\;age\;group \end{array}}$$

In this formula, standardized weight categories for animal age groups are used, implying weaners: 15 kg, finishers: 50 kg and sows and piglets: 200 kg. In the last age group, the weight of the piglets is embedded in the weight of the sows (32). DADD usage constants are not defined per product, but for each AM agent, administration route, and animal species as mg active compound per kg live animal. These values are related to the standardized use as defined in DANMAP and can vary from the actual prescribed daily dose or from the recommended dosage in the summary of the product characteristics (SPC) or from the values used to calculate VetStat ADD’s (20).

To set the overall AMU into perspective, the proportion, in thousands, of the population under treatment per day was also calculated per each of the animal age groups using the DADD per 1,000 animals per day (DAPD) indicator. As an example of the application of this indicator, 20 DAPDs stands for 2% of the population being treated with AM, on average, on any given day in 2020. It was calculated by dividing the total estimated number of kg doses (DADDs) used per year by the estimated live biomass in the age group (in tonnes, cumulated over 365 days) using the following formula:


(Formula 2) $$DAPD=\frac{\sum numberof\;kg\;doses\;\left(DADDs\right)\;\;}{estimatedlivebiomass\;}$$

where the estimated live biomass figures given per animal age group, in million tonnes, represent the number of standard animals with an estimated average weight on any given day in Denmark in 2020. For 2020, these were: sows and piglets: 9 million tonnes, weaners: 33 million tonnes, and finishers: 107 million tonnes. These figures were taken from the DANMAP database and were based on the animal census from Statistics Denmark and from the export records curated by the Danish Agriculture & Food Council.

### 2.2. Evaluation of AMU

In line with the definition set up by Aenishaenslin et al. (16), the current state of AMU was seen as an outcome of the actions to combat AMR development taken so far in Denmark, implying the last 25 years. We chose this approach because we see AMU as a driver for AMR. We did not intend to assess the impact of the individual elements of the complete Danish OH stewardship program (DANMAP). To do this, detailed investigations should be undertaken, where inspiration for these can be found in Aenishaenslin et al. (2021) (16). The AMU in Danish pig production was evaluated with respect to the risk of AMR assessing the following:

#### 2.2.1. Total AMU in the sector

The AMU for each of the three pig age groups segmented into classes, routes of administration, and treatment indications was calculated in tonnes as well as in DADD, as absolute values and as proportions of the total AMU in pigs, together with the proportion of pigs estimated as being treated on any given day in 2020. National and sectorial AMU figures, based on sales data, were also calculated in mg/PCU and interpreted in the context of the ESVAC figures for the year 2020 and compared with data from other selected countries.

#### 2.2.2. Risk related to national choice of am class in comparison with EMA recommendations

The use of the different antimicrobial AM classes, segmented according to EMA’s AMEG classification was summarized and the results interpreted according to the current risk management recommendations issued by the Danish Veterinary and Food Administration (DVFA).

#### 2.2.3. Feasibility of improving the Danish pig sector’s antimicrobial stewardship

As suggested by (33) the approach to further reduce AMR caused by AMU in Danish pig production could focus on the selection of the administration route, choice of antibiotics, as well as management improvements to lower the incidence of diseases requiring antibiotic treatment.

A visual depiction of the AMU in the sector was made, dividing the AMU by indication for treatment and administration route for each of the three age groups. This enabled us to discuss the feasibility of moving some of the AMU from oral to parenteral treatment to make further reductions and hereby improving the antimicrobial stewardship further.

Estimating the prevalence of disease in the pig sector and connecting it to the consumption of AM with precision is very difficult, hence, past works by (34) and (35) were used as to discuss this. Moreover, to get further insights and updates, we consulted two external pig veterinarians each with more than 25 years of clinical experience and associated with different major Danish veterinary advisory company.

The data analysis was performed, and the graphical outputs were produced using Microsoft Power BI® Version: 2.102.845.0.

## 3. Results and discussion

### 3.1. Total AMU in the sector

Segmenting AMU into the three different pig age groups clarified that the three age groups have different relevance for the total AMU (Table 1). Weaners registered the highest use, both in tonnes (34.4) and in DADD (201.5 million). Sows and piglets registered the second highest consumption in tonnes (22.7) but third when calculated as DADD (9.2 million). Finishers were associated with the second highest consumption when AMU was measured in DADD (37.9 million) and the third when measured in tonnes (18.8). This difference can be explained by the DADD formula, where a treatment is attributed to 50 kg individuals in the case of finishers and to 200 kg individuals in the case of sows, as described in Formula 1. By examining the consumption in DAPD units, given that this indicator is a proportion per 1,000 individuals, on average and on any given day in 2020, 1.9% of the sows were being treated with AM, as were 1.8% of the finishers and 9.2% of the weaners.

**Table 1:** Distribution of antimicrobial (AM) treatments, in tonnes and DADD units as well as the proportion of the population under treatment per day in the Danish pig sector in 2020, per pig age group category.

	Pig age group			
	Sows and piglets	Finishers	Weaners	Total
**Total use**				
Measured in tonnes	22.7	18.8	34.4	75.9
Proportion of total AMU (tonnes)	29.9%	24.8%	45.3%	100.0%
Measured in million DADD	9.2	37.9	201.5	248.6
Proportion of total AMU (DADD)	3.7%	15.2%	81.1%	100.0%
Measured in DADD per 1,000 animals per day (DAPD)	19 (1.9%)	18 (1.8%)	92 (9.2%)	

According to the 2020 ESVAC report, a 43.2% reduction was observed in the 25 countries which provided AM sales data form 2011 to 2020 (30). Even though the figures reported in ESVAC correspond to the yearly overall sales in food producing animals, given that pig production plays a major role in several European countries, it is safe to assume that the pig sector is responsible for some of these reductions (36). In the same report, Denmark’s livestock industry as a whole registered a total AMU of 37.2 mg/PCU, while the median of the 31 reporting countries was 51.9 mg/PCU (30). Our calculations show that the Danish pig sector registered an AMU of 43.3 mg/PCU. Hence, a higher value than the one registered when evaluating the consumption by the entire Danish livestock industry. The higher use in pigs is in line with a global trend, where AMU measured in mg/PCU is higher in pigs than the in other predominant livestock species which are cattle and chicken (37).

Direct international AMU comparisons based on sales data need to be interpreted carefully, as they can lead to misinterpretations (38), especially because statistics to measure AMU and denominators to estimate the animal biomass are not harmonized and different methods of data collection are applied (39). National AMU figures should also be interpreted in the context of the country’s production objectives, as these will shape the sector, by influencing the size and specialisation of the farms (40). As an example, the Danish pig sector has evolved to export weaners, which are raised in other countries, as seen in Figure 1. This means that almost half of the pigs are exported after going through the critical post weaning stage in Denmark (17). As seen in Table 1, weaners are the most treated age group, so the specialisation of the sector and consequent large proportion of very young individuals, naturally creates pressure in the overall consumption of the entire Danish sector, when measured in mg/PCU. If the exported weaners would have reached slaughter weight in Denmark, the overall AMU of the country in mg/PCU would likely have been lower than observed.

In an intensive pig production context, Denmark is usually regarded as an international example of good AMU practice (11). In a previous work by (41), the number of treatments per animal per 100 days (TI100) was estimated in a sample of heavy fattening farms, where finishers are slaughtered at 160 kg or more and destined for Parma ham production. Acknowledging the limitations of a direct comparison, the authors still highlighted a five-times higher use in Italy compared to Denmark (41). Good practices of the Danish pig sector regarding AM prescription were also underlined by Carmo et al., in a study comparing Danish and Swiss prescription patterns (42).

In conclusion, the current AMU per pig in Denmark may be considered low for finishers and for sows, given that only a small percentage of individuals is being treated, on average, on any given day during the year. Due to the specialisation of the sector, the focus for optimisation should be on the weaners, as they are responsible for the largest part of the national consumption.

### 3.2. Risk related to national choice of am class in comparison with EMA recommendations

In 2020, there was no recorded use of category A “Avoid” compounds (25). This result was expected, given that the use of these compounds is illegal in farm animals (2). In addition, no residues of these substances were found by the Danish pig meat residue monitoring program (43). Moreover, only 90 g of category B “Restrict” AM were ascribed to the entire Danish pig sector, a figure too low to be actionable in DADD units. According to the last ESVAC report, Denmark‘s livestock industry has reported the use of less than 0.01 mg/PCU each for fluoroquinolones, 3rd and 4th generation cephalosporins and polymyxins, while the median value registered by ESVAC for each of these groups was 1.1 mg/PCU, 0.2 mg/PCU and 0.8 mg/PCU, respectively (30). However, it should be emphasized that given their low defined dosage per animal kg, the use of critically important AM to human medicine tends to appear lower, when expressed in mass-based units of measure, such as the mg/PCU, when compared to dose-based units, such as the DADD (44).

**Figure 2:**
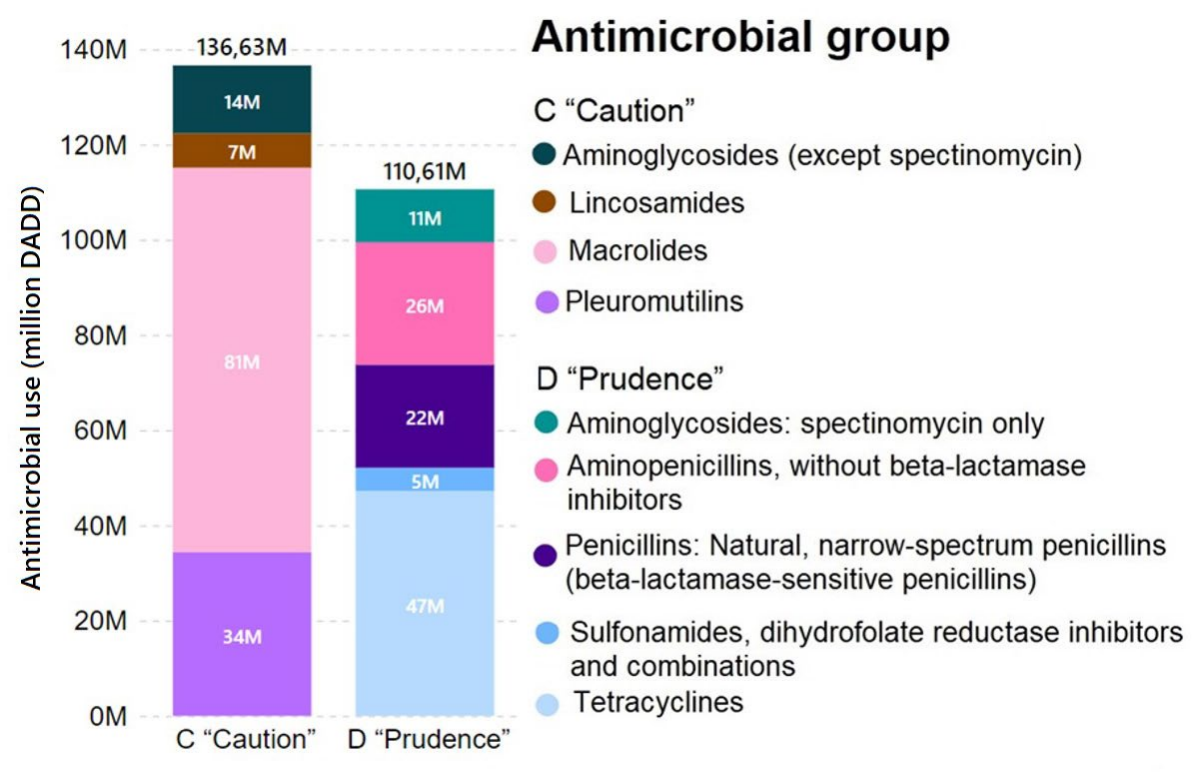
Total AM treatments in the Danish pig sector in 2020, measured as million DADD and divided according to AMEG classifications and AM class. The four AM classes of amphenicols, some penicillins (anti-staphylococcal penicillins, ß-lactamase-resistant penicillins), 3rd and 4th generation cephalosporins and polymyxins were excluded from the graph, as each class constituted less than 1% of the treatments.

Overall, there is a higher consumption of AM category C “Caution” than that of category D” Prudence” compounds in Danish pig production, as can be seen in Figure 2. The high use of category C “Caution” AMs covers macrolides (81 million DADDs) and pleuromutilins (34 million DADDs). In contrast to the AMEG classification, these AMs are 1st choice AM according to the DVFA. EMA is also more restrictive in its classification of aminoglycosides (neomycin and streptomycin) and lincosamides (lincomycin) than the DVFA (2, 26).

Regarding category D “Prudence” AMs, tetracyclines are placed in the most acceptable category by EMA but are considered a 2nd choice by DVFA and therefore associated with a weighting multiplier of 1.5 (instead of 1) in the Danish Yellow Card initiative. The decision to attribute this weighting factor is connected with the perceived role of the pig production in the emergence of livestock-associated methicillin-resistant *Staphylococcus aureus* (11, 45). This weighting likely discourages pig producers from using tetracyclines as they will more easily reach the Yellow Card limits than they would by using a non-weighted AM.

Evaluating the effect of AM exposure in the development of AMR genes is complex and each individual gene has its own dynamics in terms of emergence and dispersion (46), also studies of a representative size are required to acquire enough strength to make conclusions. Still, AMU reductions are expected to have a positive effect in reducing AMR in finisher pig gut microbiome, providing that the affected AM class is not replaced by another one (47). Risk mitigating initiatives such as the abolishment of growth promoters had a direct effect on the AMR levels detected in both pigs and broilers (48); the ban on using tylosine as a growth promoter resulted in a plummeting of the macrolide resistance in *Campylobacter* in pigs (49). Similarly, the voluntary industry ban on cephalosporin use in pig production had a significant impact in resistant *E. coli* detected at slaughter (50).

In the EU, Denmark is among those countries that report the lowest occurrence of chloramphenicol and ciprofloxacin resistance, while the occurrence of ampicillin, azithromycin, sulphonamide, trimethoprim and tetracycline resistance is comparable to the average reported by all EU Member States. Moreover, over the last 6 years, the percentage of fully sensitive *E. coli* isolates collected from caecal samples has remained approximately constant, around 46% (20).

Andersen et al., 2023 (33) estimated the quantitative effect of AMU fluctuations in Danish pig farms on the abundance of AMR genes, demonstrating that an increase or decrease in AMU is expected to cause, respectively, and increase or decrease in the abundance of AMR genes, with the stronger effects being observed over longer periods of time. The causal association between the occurrence of antimicrobial resistant bacteria in both the animal and human populations is not always clear, as shown by (51). Also, only potential associations between AMU and the emergence of AMR in humans and food-producing animals have been demonstrated (52, 53). This is partially because not many studies of the same size, as (33) have been done. In summary, for Denmark, the fact that there is effectively no use of 3rd and 4th generation cephalosporins, colistin or fluoroquinolones likely lowers the risk related to AMR development related to these substances. Moreover, the Danish legislation has led to a decrease in the use of tetracyclines since 2016, but a rise in the use of aminoglycosides, macrolides and extended-spectrum penicillin (54) in line with the recommendations by the DVFA.

### 3.3. Feasibility of improving the Danish pig sector’s antimicrobial stewardship

**Figure 3:**
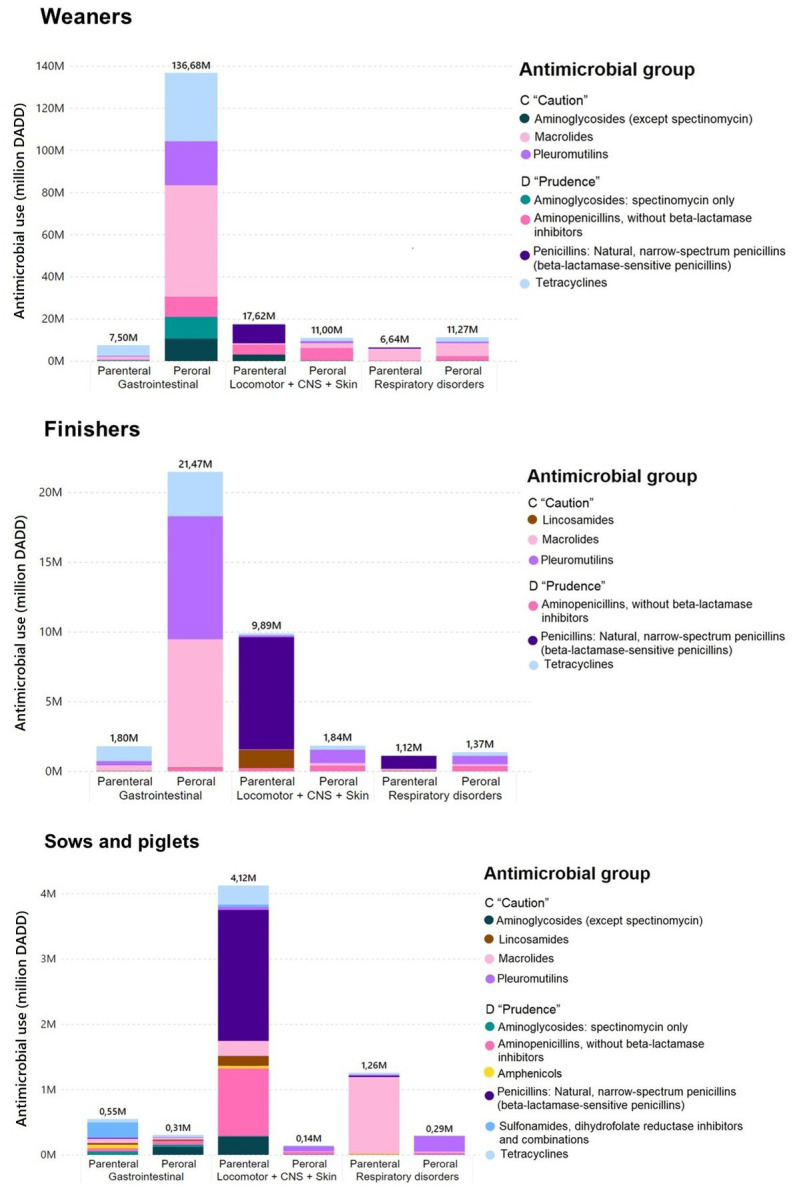
Distribution of AMU, in DADD units, in the Danish pig sector in 2020, per treatment indication, divided by AM classes, where each graph represents an age group. AM classes that constituted less than 5% of the treatments in all selected indications were excluded. Only the three most frequent treatment indications are shown. To facilitate the visualization of the antimicrobial classes consumed, 100% stacked column charts are provided in Supplementary Figure S2.

As can be seen in Table 2 and in Figure 3, the contributions of the three animal age groups to the total AMU of the pig sector differ widely, as do the treatment indications as defined by VetStat, the AM classes used and the administration route. Overall, around 99% of the pig sector AMU can be connected to three indications, among the 11 treatment indications encompassed in VetStat: “Gastrointestinal”; “Locomotor + central nervous system (CNS) + skin,” grouped together due to the low overall figures per each of the solo indications (55) and “Respiratory disorders.” Therefore, our analysis focused on these three indications. A particular emphasis was given to peroral treatments, given their major proportion and perhaps less selective usage. In Denmark, metaphylactic treatment is allowed, as in other countries (56), however, only for herds with a veterinary advisory service contract and regular use of diagnostics to confirm the diagnosis and identify resistance patterns (57). Also, in past sector interventions, major AMU reductions were connected to declines in group treatments with oral medication (58).

**Table 2:** AM treatments shown as % of DADD units used by the Danish pig sector in 2020, divided according to age category, treatment indication and administration route.

	Relative distribution (in %) of DADD according to animal age group			
	Sows and piglets	Finishers	Weaners	Total
**Treatment indication**				
Gastrointestinal	9%	62%	76%	71%
Locomotor + CNS + skin^*^	47%	31%	15%	19%
Reproduction, urogenital system	17%	0%	0%	1%
Respiratory disorders	17%	7%	9%	9%
Udder	10%	0%	0%	0%
Total	100%	100%	100%	100%
**Administration route**				
Parenteral	91%	34%	17%	23%
Peroral	9%	66%	83%	77%
Total	100%	100%	100%	100%

^*^: Treatment of joints, limbs, hooves, central nervous system and skin.

According to the two external pig veterinarians consulted, the results presented are an accurate depiction of the Danish pig sector’s routinely prescribed AM to treat the most frequent pathologies, overall speaking. Within the sows and piglets age group, most of the registered “gastrointestinal” and “respiratory” treatment indications can likely be attributed to use in piglets. Moreover, the treatment indications “locomotor + CNS + skin” and “reproduction, urogenital system and mammary gland” are mostly connected with treatment of sows.

To identify whether it would be possible to further reduce AMU in Danish pig production, the data were divided according to age group, indication, AM class and administration route. In the following, the distribution of AMU into these subgroups is discussed in relation to the types of infection causing the symptoms observed. Moreover, the focus is on the administration route, which is believed to greatly impact the effectiveness of AM treatment, while having in mind that individual treatment will most likely lead to lower AMU than group treatment (47). However, it may not be feasible to treat a higher number of pigs individually, given the logistical challenges connected to do so (56).

Table 2 shows that gastrointestinal diseases in weaners and finishers account for most of the AMU., and that the AM treatment of these infections are primarily macrolides, pleuromutilins and tetracyclines, all administered perorally. This is probably because the infections are often caused by *Lawsonia intracellularis*, *Escherichia coli* or *Brachyspira pilosicoli* (34). Macrolides and pleuromutilins can be used to treat *Lawsonia intracellularis*, and tetracyclines can be effective against both *Lawsonia intracellularis* and *E. coli* (35). For gastrointestinal disease, peroral administration of AM is commonly the preferable administration route, as the AM will work immediately in the organ of interest. As an example, it has been assessed that to threat diarrhea related *Lawsonia intracellularis* with oxytetracycline, batch treatment with oral medication is more effective than individual parental treatment (59). According to the pig veterinary specialists consulted, parenteral use of AM for gastrointestinal indications is mostly prescribed for animals that are too weak to eat or drink. Individual treatment of gastrointestinal disease is not feasible in large pig herds, where hundreds of weaners may need treatment over a short period of time. Still, the focus should be in placing ill pigs that may not be able to drink in quarantine pens and ensure individual treatments, e.g., by using injectables.

The AMEG expert advisory group also stated that to minimize AMR, individual treatments given parenteral or oral, in this order, should be preferred to oral group medication *via* drinking water and feed, given that the individual treatments are thought to have a lower general effect on AMR selection (2).

As shown by Andersen et al., (2020) parenteral AMU appear to have a high effect on resistance genes for the specific AM classes used, whereas peroral AMU tended to have a lower effect on resistance genes but for a broader range of AM classes (47). Compared to parenteral AMU, the broader impact of peroral AMU can be due to their widespread irregular during the weaner and finisher rearing periods (60, 61). Given that in Denmark, most peroral AM treatments are commonly administered in the drinking water, and weaners are routinely sorted in pens by their weight, peroral use of AM could be considerably reduced if it is targeted to as few pens or sections as necessary. This is already in practice, where a double pipe drinking water system is installed.

To treat respiratory infections in the Danish pig sector, the most used AM classes are perorally administered macrolides in weaners; penicillins are given parenterally and pleuromutilins perorally to finishers, and macrolides are given parenterally to piglets. The most common respiratory pathogens in Danish pigs are, in order of frequency according to the veterinary pig specialists consulted: *Mycoplasma hyopneumoniae*, *Mycoplasma hyorhinis*, *Glaesserella parasuis* followed by *Pasteurella multocida*, *Streptococcus suis* and *Actinobacillus pleuropneumoniae*. The last three are usually susceptible to penicillin (35), whereas *M. hyopneumoniae* is most often treated using either parenteral or peroral macrolides or peroral tetracyclines (62). Moreover, *Bordetella bronchoseptica* and *M. hyorhinis* infections are treated with either parenteral or peroral macrolides, usually soon after weaning. Because of the low weight of piglets and the way DADDs are calculated (Formula 1), with sows and piglets being in the same group, relatively few DADDs can be used to treat several animals, considering the DANMAP dosage per kg of body weight. Peroral administration of AM for respiratory indications is mostly used for metaphylactic treatments.

For locomotor, CNS and skin indications, the most prescribed AM classes were parenterally administered penicillins and aminopenicillins, in all three age groups. Infections related to these indications are mostly due to *Streptococcus suis* and *Erysipelothrix rhusiopathiae*, which tend to be susceptible to ß-lactams, and *Mycoplasma hyosynoviae* that can be successfully treated with macrolides or tetracyclines (35, 63). Peroral administration of AM for locomotor, CNS and skin indications is most likely used for metaphylaxis.

In a cross-sectional study conducted in four European countries, 227 farrow-to-finish pig farms were comparing regarding AMU by age category, antimicrobial class and administration route (64). According to this study, Sweden stood out with a comparatively low AMU in weaners, and an overall predominance of parenteral treatments (87%). These figures, along with the Swedish livestock sector registering an AMU of 11.1 mg/PCU (30) in 2020, and a pig sector internationally praised for its actions on herd treatments (65), where individual treatments accounted for 80% of the AMU (66) suggest that Sweden can be mentioned as an example of a country that has managed to reduce AMU to a larger extent than many other countries. The Swedish legislation specifies that group treatment can only be prescribed on a case-by-case basis, after the implementation of a written and compulsory disease control program based upon a systematic analysis of the disease issue (67). Likewise, the national guidelines state that group treatment of post-weaning diarrhea should only be considered if more than 25% of the pigs in a litter are affected (50). However, Swedish pig herds are smaller than Danish pig herds, in general, which makes it easier to treat animals individually than in Denmark. The mean Swedish sow and fattening farms have 185 sows and 945 pigs, respectively (68); in contrast, the median Danish sow and fattening farms have around 500 to 599 sows and 2,000 to 2,999 pigs, respectively (17). Since 2014, in Denmark, group treatment prescriptions require laboratory diagnosis and the elaboration of an action plan aiming to reduce the need for group treatments. Furthermore, the prescription’s effect on the herd health must be followed up in the farm’s trimestral veterinary advisory report together with an evaluation conducted by the farm veterinarian justifying the need to continue the treatments, 3 months after the first assessment (69).

In conclusion, the extended use of the peroral AM administration route in Danish pigs is mostly related to treatment of gastrointestinal disease in weaners. This makes sense from the veterinary and economic points of view, due to feasibility when treating numerous animals simultaneously. Peroral AMU is higher than parenteral AMU for gastrointestinal indications for weaners and finishers. For all the other indications, most of the use is parenteral, implying individual treatment that should result in less AMU than in the case of peroral treatment.

The last 10 years’ reductions and shifts achieved in the use of AMs of critical importance in European pig production suggest that further reductions in AMU in the pig sector are possible (36). The question is which initiatives will achieve this. According to the two external pig field veterinarians consulted, to further decrease AMU in the Danish pig sector, the focus should be on disease prevention and animal health promotion. This could be achieved by providing better quality feed, increasing group vaccination, and improving external and internal biosecurity. This is in line with international pig specialists, who ranked these measures among the most promising to promote responsible AMU, taking into consideration the measures’ combined effectiveness, feasibility and return on investment (70). Numerous studies have shown that targeted use of vaccines in animal populations can lead to a significant decrease in the consumption of AMs (71). Moreover, the two veterinary pig specialists recommended that the need of vaccines should be considered individually for each farm, especially vaccines against the bacteria *E. coli*, *Mycoplasma* spp., *Lawsonia intracellularis* and *Actinobacillus pleuropneumoniae* and the viruses PCV2, influenza and PRRS. Improvement of the overall animal health combined with prevention of the outset of primary or secondary infections can act as effective measure. This is despite the study by Kruse et al. (72) showing that vaccine use was not related to lower AMU, because vaccines may have been used to handle existing disease problems, and hence, reverse causality was observed (72). Moreover, new and possibly more effective vaccines have been developed since the study by Kruse et al. (72) was published. Vaccination cannot be considered a stand-alone measure but should be part of a multi-action plan also involving external and internal biosecurity. Danish farmers often state the cost of vaccination as a major limitation; despite this, vaccines sales have been increasing in Denmark, as can be seen in Supplementary Figure S1.

Denmark’s specific pathogen free (SPF) system aims to avoid the introduction of specific pathogens into pig herds, and has led to eradication of swine dysentery, an infection for which high AMU is ascribed (11). Other national eradication programs are being considered in Denmark, with that against PRRS virus being recently initiated (73). An initiative that could result in significant reduction of AMU at the national level.

To further promote responsible AMU, the Danish Agriculture & Food Council has released a manual on good antibiotic practices, with simple and easy to follow guidelines on the prevention and diagnosis of diarrhea in weaners and finishers and the handling of antibiotic treatments (6). The manual is promoted among farmers and updated when necessary or as new relevant knowledge arises.

The Yellow Card permit limits were originally defined as twice the average use among the country’s pig farms. Further AMU reduction targets should be accompanied by careful animal health and welfare assessments (57) to ensure that animals are being treated “As little as possible, but as often as necessary.” Finally, it was found that the VetStat monitoring system is working effectively, as it is set up to present AMU in detail, which includes age group, indication, AM and administration route. This allows the identification of high use segments, which potentially could lead to implementation of targeted interventions. Still, one issue to consider when operating a Yellow Card-like system is that permitted limits could be interpreted as acceptable limits, which is not the intention of the system.

Sanders et al. (2020) analysed the multiple strategies followed concerning the essential system design elements and management processes of AM stewardship initiatives, based on farm-level AMU, demonstrating that there is no widely accepted approach to implement such initiatives (44). The decisions made in Denmark should be considered in the context of the country and may not be universally applicable. As an example, in Italy the ClassyFarm system benchmarks farms by comparing their usage either at age group or herd level to median of all farms and classifies them according to quartiles. However, a similar approach to the Danish yellow card has been followed by a private system in the Czech Republic (Q VET pigs) and two quality assurance system in Switzerland (SuisSano and Safety+) also define a multiplication factor for the use of high priority critically important AM (74).

## 4. Conclusion

After more than 25 years of AMU stewardship-related interventions in the Danish pig sector, the sector’s AMU can be considered responsible in an intensive livestock production context. There is no use of AM in the category A “Avoid” and a minuscule use of category B” Restrict.” Most peroral use is related to weaners suffering from gastrointestinal infections. To further reduce the pig sector’s AMU, a further shift from section to pen or individual treatments should be considered. To ensure prudent use of AM, enhanced focus should be on the prevention of disease and the promotion of animal health through the rearing of more robust pigs, use of better feed, more vaccines and increased biosecurity.

## Data availability statement

The data analyzed in this study is subject to the following licenses/restrictions: The data set may be consulted under the terms defined by the Danish Program for surveillance of antimicrobial consumption and resistance in bacteria from food animals, food and humans. Requests to access these datasets should be directed to MS, marsan@food.dtu.dk.

## Author contributions

PM, MS, and LA took equal leadership in all steps of the study, from the conception to the design of the work, and drafting the first versions of the manuscript. JN-R provided a critical external view to the study. BH supervised the data analysis and figure production. EN contributed with clinical and legal expertise. All authors contributed to the article and approved the submitted version.

## Funding

This work was partly undertaken as part of CoEvalAMR Phase 2, which is funded by the JPIAMR and led by the Faculty of Veterinary Medicine at the University of Montreal, receiving funding from the Canadian Institutes for Health Research.

## Conflict of interest

LA and EN work for two organisations which provide advice to farmers and meat producing companies.

The remaining authors declare that the research was conducted in the absence of any commercial or financial relationships that could be construed as a potential conflict of interest.

## Publisher’s note

All claims expressed in this article are solely those of the authors and do not necessarily represent those of their affiliated organizations, or those of the publisher, the editors and the reviewers. Any product that may be evaluated in this article, or claim that may be made by its manufacturer, is not guaranteed or endorsed by the publisher.
