# Supplementary material for: Characterisation of antimicrobial usage in Danish pigs in 2020
Source: Front Vet Sci. 2023 Apr 25;10:1155811. doi: 10.3389/fvets.2023.1155811 (PMC10167271; doi:10.3389/fvets.2023.1155811)
Supplement: Supplementary file 1 [file Data_Sheet_1.docx]

Characterisation of antimicrobial usage in Danish pigs in 2020

Pedro Moura, Marianne Sandberg, Birgitte Borck Høg, João Niza-Ribeiro, Elisabeth Okholm Nielsen, Lis Alban*

***** Corresponding Author: lia@lf.dk

**Supplementary material**


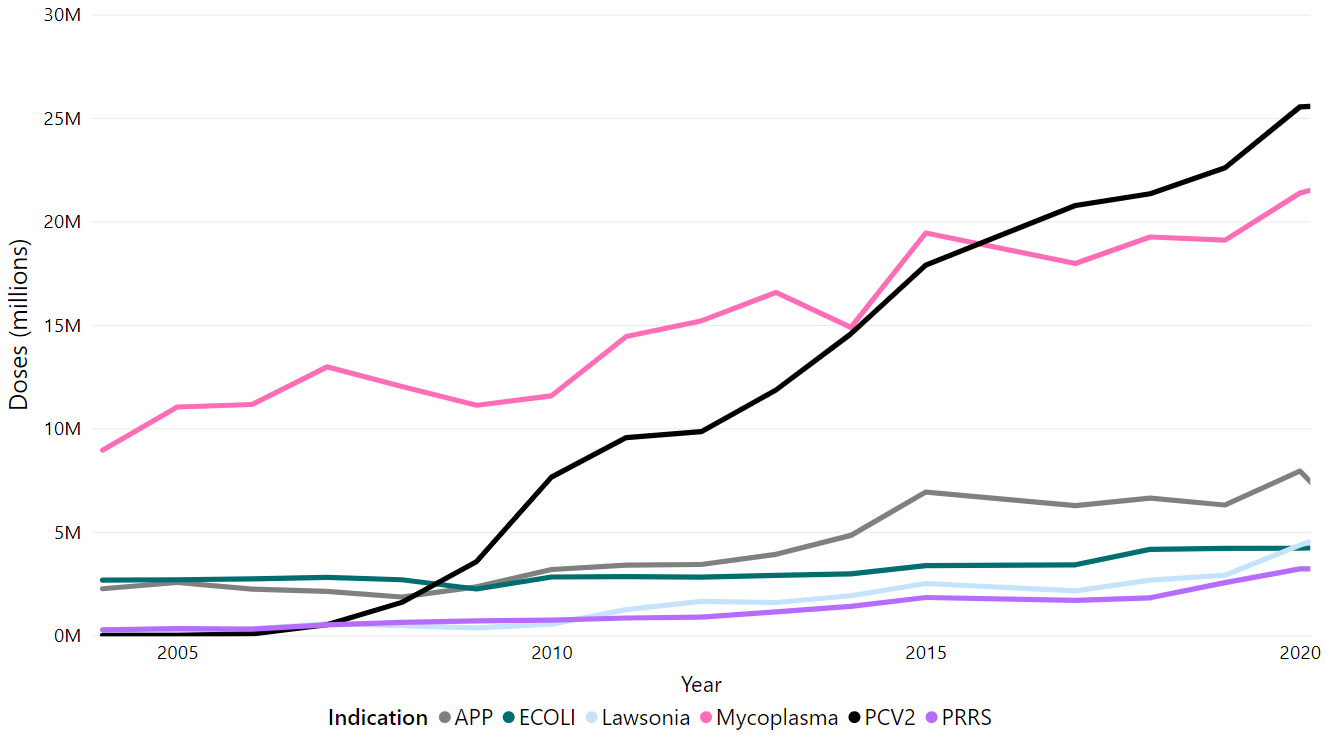


**Figure S1**: Sales of specific vaccines for Danish pig herds in the period from 2005 to 2020 Source: VetStat; data from 2016 were not available. Vaccination against: APP = *Actinobacillus pleuropneumoniae*, ECOLI = *Escherichia coli*, Lawsonia = *Lawsonia intracellularis*, *Mycoplasma* = *Mycoplasma hyopneumoniae*, PCV2 = Porcine circovirus, PRRS =  Porcine reproductive and respiratory syndrome.

**Table S1:** Antimicrobial substances recommendations, as defined by DVFA’s groups^a^ for pig production and EMA’s categories^b^

| **Antimicrobial substance** | **DVFA** | **EMA** |
| --- | --- | --- |
| Florfenicol | Group 1 | C |
| Lincomycin | Group 1 | C |
| Macrolides | Group 1 | C |
| Neomycin | Group 1 | C |
| Penicillin, narrow-spectrum | Group 1 | D |
| Penicillin, broad-spectrum | Group 1 | D |
| Pleuromutilins | Group 1 | C |
| Sulphonamides | Group 1 | D |
| Spectinomycin | Group 1 | D |
| Streptomycin | Group 1 | C |
| Apramycin | Group 2 | C |
| Gentamicin | Group 2 | C |
| Tetracycline | Group 2 | D |
| 3rd and 4th generation cephalosporins | Group 3 | B |
| Colistin | Group 3 | B |
| Fluoroquinolones | Group 3 | B |

^a^ Group 1: First line antimicrobials to be used when a veterinarian has determined that AM treatment is necessary; Group 2: Alternative AM when first line AM are not effective; Group 3: These AMs should not be used in pigs
^b^ Category A: “Avoid”; Category B: ”Restrict”; Category C: ” Caution”; Category D: ”Prudence”.


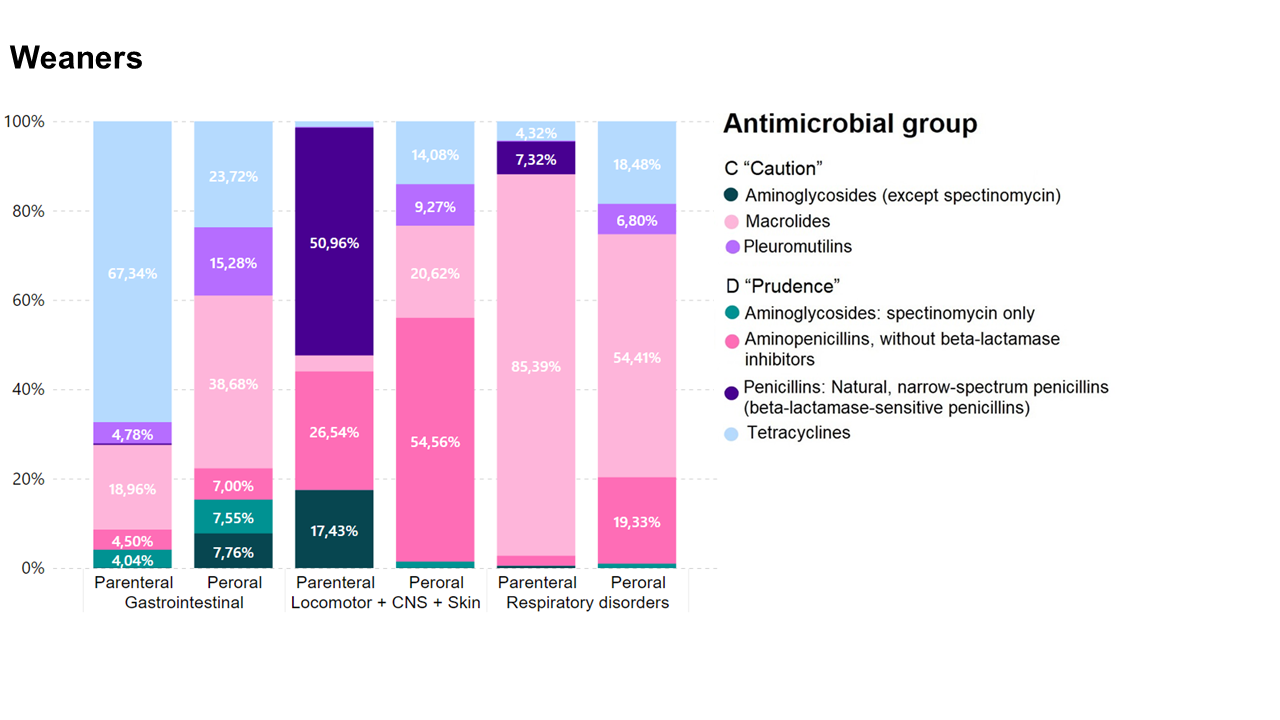

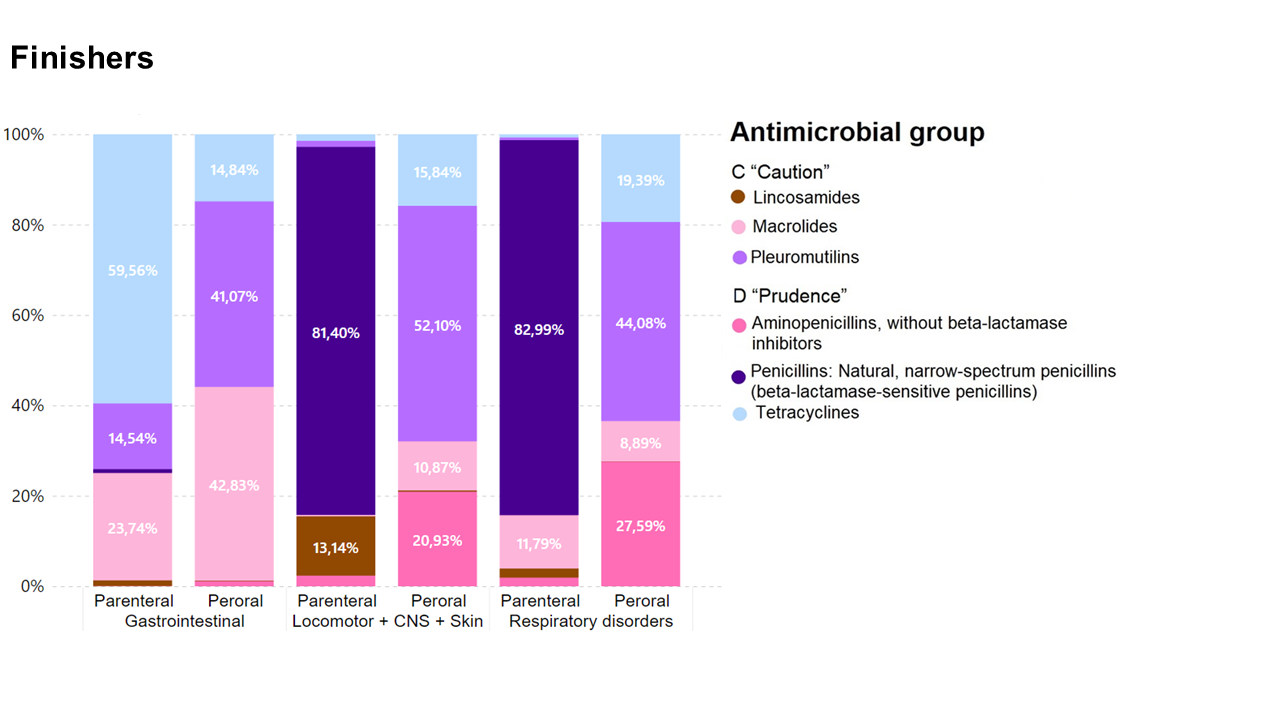

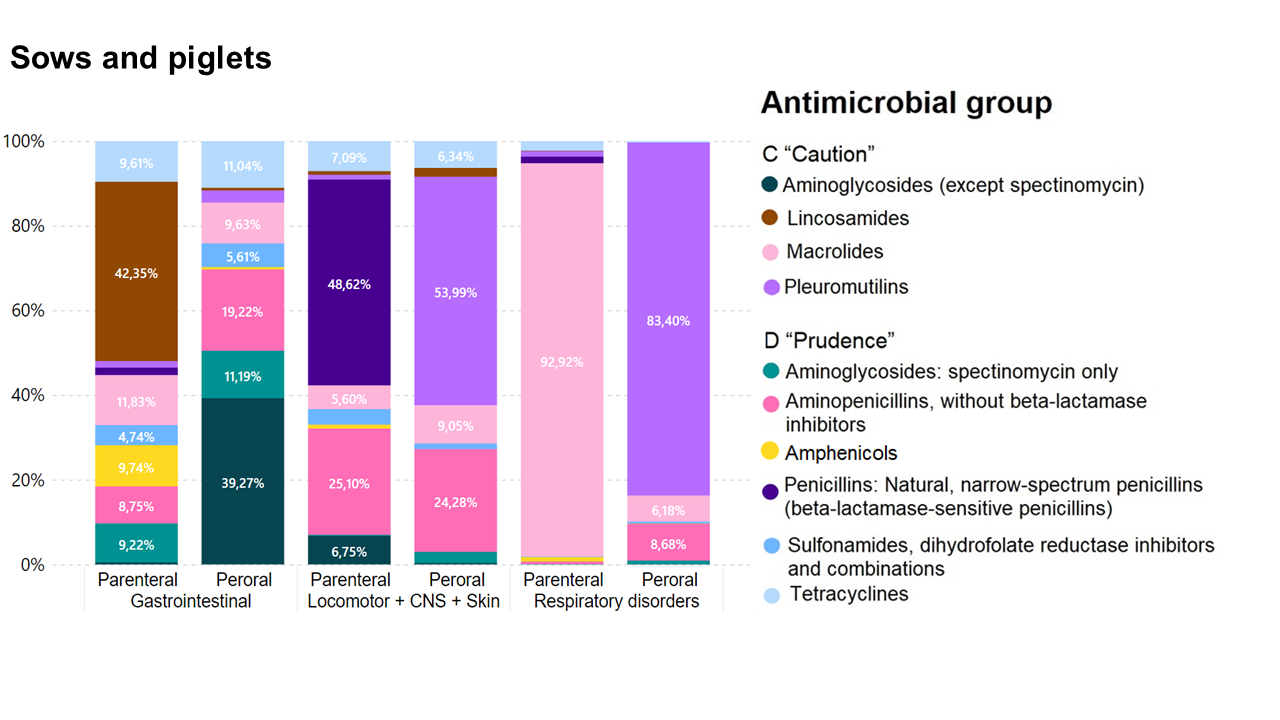


**Figure S2** : Distribution of AMU, in DADD units, in the Danish pig sector in 2020, per treatment indication, divided by AM classes, where each graph represents an animal age group. AM classes that constituted less than 5% of the treatments per indication were excluded. Only the three most frequent treatment indications are shown.
